# Draft Genome Sequence of *Lactococcus lactis* Subsp. *cremoris* WA2-67: A Promising Nisin-Producing Probiotic Strain Isolated from the Rearing Environment of a Spanish Rainbow Trout (*Oncorhynchus mykiss,* Walbaum) Farm

**DOI:** 10.3390/microorganisms10030521

**Published:** 2022-02-28

**Authors:** Javier Feito, Diogo Contente, Manuel Ponce-Alonso, Lara Díaz-Formoso, Carlos Araújo, Nuria Peña, Juan Borrero, Beatriz Gómez-Sala, Rosa del Campo, Estefanía Muñoz-Atienza, Pablo E. Hernández, Luis M. Cintas

**Affiliations:** 1 Grupo de Seguridad y Calidad de los Alimentos por Bacterias Lácticas, Bacteriocinas y Probióticos (SEGABALBP), Sección Departamental de Nutrición y Ciencia de los Alimentos, Facultad de Veterinaria, Universidad Complutense de Madrid, Avda, Puerta de Hierro, s/n., 28040 Madrid, Spain; j.feito@ucm.es (J.F.); diogodas@ucm.es (D.C.); lardia01@ucm.es (L.D.-F.); carlos.alex.araujo@gmail.com (C.A.); nuriapen@ucm.es (N.P.); jborrero@ucm.es (J.B.); ehernan@ucm.es (P.E.H.); lcintas@ucm.es (L.M.C.); 2 Servicio de Microbiología, Hospital Universitario Ramón y Cajal & Instituto Ramón y Cajal de Investigación Sanitaria (IRYCIS), Ctra. Colmenar Viejo, km. 9, 100., 28034 Madrid, Spain; lugonauta@gmail.com (M.P.-A.); rosacampo@yahoo.com (R.d.C.); 3 APC Microbiome Ireland, University College Cork, T12 K8AF Cork, Ireland; beatriz.gomezsala@teagasc.ie; 4 Teagasc Food Research Centre, Moorepark, Fermoy, P61 C996 Cork, Ireland

**Keywords:** aquaculture, probiotics, lactic acid bacteria, bacteriocins, nisin Z

## Abstract

Probiotics are a viable alternative to traditional chemotherapy agents to control infectious diseases in aquaculture. In this regard, *Lactococcus lactis* subsp. *cremoris* WA2-67 has previously demonstrated several probiotic features, such as a strong antimicrobial activity against ichthyopathogens, survival in freshwater, resistance to fish bile and low pH, and hydrophobicity. The aim of this manuscript is an in silico analysis of the whole-genome sequence (WGS) of this strain to gain deeper insights into its probiotic properties and their genetic basis. Genomic DNA was purified, and libraries prepared for Illumina sequencing. After trimming and assembly, resulting contigs were subjected to bioinformatic analyses. The draft genome of *L. cremoris* WA2-67 consists of 30 contigs (2,573,139 bp), and a total number of 2493 coding DNA sequences (CDSs). Via in silico analysis, the bacteriocinogenic genetic clusters encoding the lantibiotic nisin Z (NisZ) and two new bacteriocins were identified, in addition to several probiotic traits, such as the production of vitamins, amino acids, adhesion/aggregation, and stress resistance factors, as well as the absence of transferable antibiotic resistance determinants and genes encoding detrimental enzymatic activities and virulence factors. These results unveil diverse beneficial properties that support the use of *L. cremoris* WA2-67 as a probiotic for aquaculture.

## 1. Introduction

Aquaculture is a cornerstone in the food supply chain and the fastest-growing food-producing sector worldwide [1,2,3]. The foreseeable growth in human population, which could reach, according to projections, 9.8 billion people by 2050, will increase the demand for food and protein sources [4]. In this respect, aquaculture is regarded as a key alternative to supply this growing demand [3]. Nevertheless, for aquaculture to meet this demand, intensification, and expansion, it will need to face challenges such as disease outbreaks, the emergence and re-emergence of bacterial ichthyopathogens, and antimicrobial resistant bacteria [1,5].

The widespread abusive and erroneous use of antibiotics and other veterinary drugs in aquaculture has been to some extent underrated in the past. Nonetheless, aquatic environments and the intensive nature of aquaculture are not only prone to support the thriving of diverse pathogenic bacteria, but they can also act as reservoirs for antimicrobial-resistant bacteria and antimicrobial resistance transferable genes. The residues and sedimentation of the uneaten, non-absorbed antimicrobial compounds administered by medicated feeds, and their metabolites, pose a serious threat of contamination to the surrounding ecosystems. Additionally, there is an imminent hazard to the human health through direct contact with contaminated facilities, or even through foodborne infections, considering that a significant number of ichthyopathogens (e.g., *Lactococcus garvieae*, *Streptococcus agalactiae*, *Vibrio* spp.) are also zoonotic agents [1,5,6]. In this regard, novel and complementary strategies for disease control in aquaculture have emerged, including vaccines, probiotics, prebiotics/symbiotics, parabiotics, postbiotics, bacteriophages, and immunostimulants [1,7,8,9].

Although there are no specific guidelines for the evaluation and selection of probiotics intended for aquaculture, there are general recommendations for their evaluation. These recommendations are based upon safety assessments of microorganisms, both used in food and feed, taxonomical identification, and for instance, resistance to antibiotics of clinical importance for human medicine. In this regard, recently, the European Food Safety Authority (EFSA) established the whole-genome sequence (WGS) of a bacterial strain as a requisite for its future use in the food chain and stated the requirements for WGS analysis of microorganisms intentionally used for this purpose [10,11]. To date there is only one probiotic authorized for use in aquaculture by the European Union, which is *Pediococcus acidilactici* CNCM I-4622 (formerly named MA18/5M) [7,8,12].

Lactic acid bacteria (LAB) is the most representative group of bacteria proposed as probiotics for aquaculture. There is a growing interest in the use of LAB as probiotics in aquaculture for reasons such as the general classification of most LAB as Generally Recognized as Safe microorganisms (GRAS) and/or having Qualified Presumption of Safety (QPS) status, and their current use as probiotics for both human and animal health. Moreover, LAB have been assessed as probiotics due to their antimicrobial properties, such as the production of organic acids and ribosomally synthesized antimicrobial peptides referred to as bacteriocins [7,8]. Bacteriocin production is a common trait of LAB, and many bacteriocinogenic strains have been characterized, displaying antimicrobial activity against a wide range of Gram-positive bacteria, and, to a lesser extent, Gram-negative bacteria, including spoilage and foodborne pathogens. Their spectrum of antimicrobial activity has attracted great interest for their potential as food preservatives and as therapeutical agents in both human and veterinary medicine [13]. In this regard, *Lactococcus lactis* subsp. *cremoris* WA2-67, a strain isolated from the rearing environment of a Spanish rainbow trout (*Oncorhynchus mykiss,* Walbaum) farm, has been previously assessed for its potential as a probiotic for aquaculture [14,15]. Interestingly, *L. cremoris* WA2-67 has previously demonstrated several probiotic features, such as survival in freshwater, resistance to fish bile and low pH, hydrophobicity, and broad and strong antimicrobial activity against many ichthyopathogens, including *Lactococcus garvieae*, which is a major pathogen in the rainbow trout aquaculture. The antimicrobial activity exerted by *L. cremoris* WA2-67 is due to the production of a bacteriocin identified as nisin Z (NisZ), being the first description of a NisZ-producing *L. lactis* strain isolated from an aquatic environment. Moreover, the safety of *L. cremoris* WA2-67 was demonstrated by the absence of antibiotic resistance genes, production of biogenic amines, hemolysin and gelatinase, and its inability to degrade gastric mucin or deconjugate bile salts [15]. Additionally, in vivo challenge tests demonstrated the ability of *L. cremoris* WA2-67 to protect rainbow trout against bacterial infection by *L. garvieae*, demonstrating, for the first time, the role of a bacteriocin (NisZ) as an effective weapon in fish farm conditions [16].

Considering the probiotic potential exhibited by *L. cremoris* WA2-67, EFSA requirements for the evaluation of microorganisms intentionally used in the food chain and the need to gain deeper insight into the safety and probiotic properties and their respective genetic foundations, the draft genome sequence of this bacteriocinogenic strain was determined. Then, we performed in silico analysis through use of diverse databases, tools, and software [17,18,19,20].

## 2. Materials and Methods

### 2.1. Growth Conditions and Genomic DNA Isolation

*L. cremoris* WA2-67 was cultured in de Man, Rogosa and Sharpe (MRS) agar (1.5% *w*/*v*) plates (Oxoid, Basingstoke, UK) at 30 °C for 16 h.

For genomic DNA isolation, the NZY Microbial gDNA Isolation Kit (NZYTech, Lisbon, Portugal) was used. Briefly, cells were harvested by centrifugation, and then the pellet was resuspended in 100 µL of elution buffer. The resulting mixture was transferred into a NZYSpin Microbial Bead Tube, where 40 µL of buffer NML and 10 µL of proteinase K were added. For the sample lysis, this new resuspension was applied to a TissueLyser II (Qiagen, Hilden, Germany). Then, for DNA binding, 600 µL of buffer NML were added and briefly vortexed. After centrifugation at 11,000× *g* for 30 s, the supernatant was transferred into a NZYSpin Microbial Column for a new centrifugation under the same conditions. The silica membranes of the column were washed by adding 500 µL of buffer NMW1, followed by centrifugation. Then, 500 µL of buffer NMW2 were added, and the mixture centrifuged. In order to guarantee the removal of the washing buffers the column was centrifuged once again at 11,000× *g* for 30 s. For DNA elution, 100 µL buffer NME were added to the column, and after incubation at room temperature for 1 min, recentrifuged at 11,000× *g* for 30 s. After this point, the eluate was stored at −20 °C for further use.

### 2.2. Draft Genome Sequencing, Assembly, and Mapping

The Nextera XT library preparation kit (Illumina, San Diego, CA, USA) and the Microlab STAR automated system for liquid manipulation (Hamilton, Reno, NV, USA) were used to obtain DNA libraries. Libraries were quantified with a KAPA library quantification kit (Roche, Basel, Switzerland). Subsequently, whole-genome sequencing was performed in “Servicio de Secuenciación y Bioinformática de FISABIO” (Valencia, Spain) using a MiSeq system (Illumina), with the 250-bp paired-end sequencing protocol. Reads were analyzed with Trimmomatic v.0.38, for adaptor removal [21]. Reads with average Phred scores of ≥15 and lengths of >36 bp were de novo assembled using SPAdes v.3.12.0 [22]. The quality of the assembled sequences was assessed using the QUAST v.5.0.2 tool [23]. Coding DNA sequences (CDSs) were predicted and annotated using the NCBI Prokaryotic Genome Annotation Pipeline (PGAP) [24]. Finally, a genome map of *L. cremoris* WA2-67 was generated using CGView Server (http://cgview.ca/, accessed on 25 December 2021) [25].

### 2.3. Bioinformatic In Silico Analysis

#### 2.3.1. Identification

Two different approaches were used for species and subspecies identification. First, the SpeciesFinder v.2.0 tool (https://cge.cbs.dtu.dk/services/SpeciesFinder/, accessed on 25 December 2021) was used for a more traditional species prediction based on the complete sequence of the *16S rRNA* gene [26]. Then, the identification was checked using KmerFinder v.3.0.2. (https://cge.cbs.dtu.dk/services/KmerFinder/, accessed on 25 December 2021), a tool that predicts bacterial identity based on the number of concurrent k-mers (namely, 16-mers) between the assembled genome and those genomes present in the database [27].

#### 2.3.2. Probiotic Traits

A manual prospection of CDSs was carried out by using the online server Rapid Annotation using Subsystem Technology (RAST) and SEED v.2.0 (http://rast.nmpdr.org/, accessed on 25 December 2021). The SEED is an online database that integrates updated genomic data, being a reliable tool to predict gene functions, metabolic pathways, and other bioinformatic data [28]. Amongst the probiotic traits analyzed were factors related to adhesion and aggregation, vitamin biosynthesis, amino acids metabolism, production of lactic acid, active metabolism, enzyme production for food digestion, stress, and host gastrointestinal tract adaptations.

#### 2.3.3. Bacteriocin Production

For bacteriocin, ribosomally synthesized and post-translationally modified peptides mining, the assembled genome in FASTA format, excluding contigs with a length under 3000 bp, was uploaded and analyzed under default settings in the online webserver BAGEL v.4.0 (http://bagel4.molgenrug.nl/, accessed on 25 December 2021) [29].

#### 2.3.4. Mobile Genetic Elements (MGE)

Both types of MGE were searched, that is, intracellular MGE, namely insertion sequences (IS), and intercellular MGE, such as plasmids and prophages.

##### Insertion Sequences (IS)

IS constitute one of the largest groups of MGE, or sometimes referred to as mobilome. In this regard, the ISfinder database (https://www-is.biotoul.fr/index.php, accessed on 25 December 2021) was used in order to analyze the presence of bacterial IS in the genome of *L. cremoris* WA2-67 [30].

##### Plasmids

The PlasmidFinder web-tool v.2.0.1. (https://cge.cbs.dtu.dk/services/PlasmidFinder/, accessed on 25 December 2021) was used to detect the presence of plasmids in the WGS of *L. cremoris* WA2-67. This web-tool allows the detection of replicons in the WGS and assembles them under lineages [31,32]. Settings were adjusted for a threshold for minimum percentage identity of 90%, and with a minimum coverage of 60%.

##### Prophages

For the detection of prophage regions, the Prophage Hunter web-service (https://pro-hunter.genomics.cn, accessed on 25 December 2021) was employed. The Prophage Hunter matches similarities within a phage library, scores the probability of a prophage being active within a bacterial genome, annotates the function of phage proteins, and identifies phylogenetically related phages [33].

#### 2.3.5. CRISPR/CRISPR-Cas

The CRISPRCasFinder online program v.1.1.2. (https://crisprcas.i2bc.paris-saclay.fr/CrisprCasFinder/Index, accessed on 25 December 2021) was employed to predict clustered regularly interspaced short palindromic repeats (CRISPR) and CRISPR-associated genes (*cas*) [34].

#### 2.3.6. Transferable Antibiotic Resistances

A BLASTn search was performed against the ResFinder tool v.4.1. database (https://cge.cbs.dtu.dk/services/ResFinder/, accessed on 25 December 2021) to search for acquired genes encoding antimicrobial resistances. The ResFinder is a tool that identifies acquired genes or chromosomal mutations that mediate antimicrobial resistances in total or partial bacterial DNA [35].

#### 2.3.7. Virulence Factors

Likewise, a BLASTn search was run in search of matchings using the VirulenceFinder v.2.0.3 database (https://cge.cbs.dtu.dk/services/VirulenceFinder/, accessed on 25 December 2021), in order to find and predict genes encoding for bacterial virulence factors [36].

Additionally, a prediction to assess the virulence and pathogenicity towards human health was performed using the PathogenFinder v.1.1. webserver (https://cge.cbs.dtu.dk/services/PathogenFinder/, accessed on 25 December 2021) [37].

## 3. Results and Discussion

### 3.1. Draft Genome Sequencing, Assembly, and Mapping

**Figure 1:**
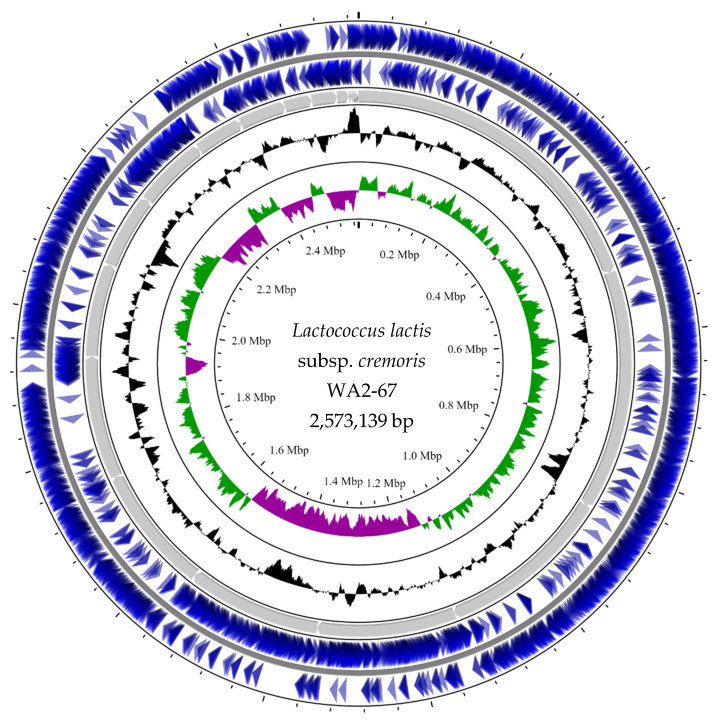
*L. cremoris* WA2-67 genome map generated using the CGView Server (http://cgview.ca/, accessed on 25 December 2021) [25], showing a full view of the genome. The blue arrows represent the CDSs and the gray arrows the contigs. The black plot shows GC content, while the green and magenta plot shows CG skew + and −, respectively.

The draft genome of *L. cremoris* WA2-67 consists of 30 contigs (2,573,139 bp), with a G + C content of 35.4%, and N50 and L50 values of 214,342 and 4, respectively. The total numbers of CDSs and RNAs were 2493 and 65, respectively. Figure 1 shows the genome map generated in CGView Server.

### 3.2. Bioinformatic In Silico Analysis

#### 3.2.1. Identification

The species prediction based on the complete sequence of the *16S rRNA* gene, performed by SpeciesFinder v.2.0 tool, identified the strain as *Lactococcus lactis.* Additionally, prediction using KmerFinder v.3.0.2. revealed the most likely subspecies identification to be *L. lactis* subsp. *cremoris.* In this regard, the strains present in the database with the highest matching scores (i.e., number of matching kmers) were *Lactococcus lactis* subsp. *cremoris* KW2 (accession number NC_022369.1) and *Lactococcus lactis* subsp. *cremoris* NZ9000 (accession number NC_017949.1), with 63,348 and 7610 matching kmers, respectively. These bioinformatic tools confirmed our previous results obtained by partial *16S rRNA* gene sequencing, identifying our strain as *Lactococcus lactis* subsp. *cremoris* WA2-67 [14,15].

#### 3.2.2. Probiotic Traits

Amongst the probiotic traits, analyzed through RAST, were putative genes involved in adhesion and aggregation, vitamin biosynthesis, amino acids metabolism, production of lactic acid, active metabolism, enzyme production involved in food digestion, and stress and host gastro-intestinal tract adaptation (Table S1).

One of the most important traits for a potential probiotic strain is the ability to adhere to the gastro-intestinal tract of the host. In this regard, the genome analysis of *L. cremoris* WA2-67 identified genes encoding enolase, fibronectin-binding protein, exopolysaccharide (EPS) biosynthesis protein, triosephosphate isomerase, and sorties A (LPXTG). Enolase is a protein that promotes the adherence of the strain to the gastro-intestinal tract of the host, while the fibronectin-binding protein allows the bacterium to adhere to fibronectin of the host. Additionally, the production of EPS by probiotic strains is linked with the adherence to intestinal mucus, while proteins with the LPXTG-type anchor, as the identified sortase, are associated both with cell surface localization and interaction with peptidoglycans [38]. Another desirable probiotic trait is the production of micronutrients, such as vitamins. Vitamins are not only necessary for fish growth and development but have also been used as immunostimulants in aquaculture [39,40]. The RAST analysis identified several genes involved in the production of several B-group vitamins, such as thiamine (vitamin B_1_), riboflavin (vitamin B_2_), pyridoxin (vitamin B_6_), biotin (vitamin B_7_), and folate (vitamin B_9_). The importance of B-group vitamins for aquaculture and fish has been previously assessed and demonstrated. For instance, thiamine (vitamin B_1_) was linked with a reduction of dead and deformed fry in thiamine-supplement fish [41]. Moreover, thiamine (vitamin B_1_) deficiency was associated with early mortality syndrome in salmonids, such as lake trout (*Salvelinus namaycush*) [42]. Other B-group vitamins, such as biotin (vitamin B_7_), are also regarded as essential for growth, development, welfare, and reproduction parameters in fish [43]. Likewise, folate (vitamin B_9_) not only is regarded as an essential micronutrient for teleosts, such as several salmonids, but it is also associated with growth rate, cellular proliferation, and embryogenesis requirements for cold-water species [44].

The RAST analysis also allowed us to identify multiple genes involved in the metabolic pathways of numerous amino acids, such as threonine, tryptophan, methionine, leucine, lysine, cysteine, histidine, and arginine. Proteins and amino acids are fundamental for fish nutrition, and such deficiencies have demonstrated impact on the immune system, namely negatively affecting the susceptibility of fish to infectious diseases [45,46]. For instance, tryptophan is a metabolic precursor of several compounds such as serotonin, melatonin, and niacin (vitamin B_3_). Nevertheless, as an essential aromatic amino acid, tryptophan has to be supplied via feed. For this reason, the aquaculture industry has tried for many years to optimize the levels of tryptophan in feed, as it takes part in multiple physiological functions, such as behavior modulation, antioxidant, stress, and immune responses [47]. Similarly, arginine is also an essential amino acid that is mostly provided to fish via diet. In fish, arginine is involved in various functions, such as innate immune defense by stimulating nitric oxide (NO) production by macrophages, ammonia detoxification, insulinotropic effect, somatotropic axis, growth performance, and antioxidant action [46]. On the other hand, the role of histidine in fish physiology has been previously linked with reproduction, spawning, and embryogenesis parameters, such as fecundity and fertilization rates, egg viability, hatching rates, larval survival, egg protein content, and egg and larval size [48].

LAB attract great interest from different research areas and industries mostly due to lactic fermentation, whose end-product is lactate, which exhibits antimicrobial activity [49]. Lactic acid can be found in both d and l enantiomer forms, depending on the genetic determinants encoding d-lactate or l-lactate dehydrogenase, respectively [50,51]. The RAST analysis of *L. cremoris* WA2-67 identified the presence of only l-lactate dehydrogenase (Table S1), thus predicting that this strain produces the enantiomer l-lactate but not d-lactate, which is a desirable feature for a probiotic candidate since d-lactate acidosis, under certain circumstances, may lead to animal poor appetite and retarded growth [52].

As mentioned above, due to the enormous interest of LAB, not only as probiotics per se, but also as starter microorganisms in the production of fermented foods, their ability to resist and cope with temperature changes is a feature of the utmost technological importance, given the different temperatures they will undergo during the processing and subsequent refrigeration or frozen storage [53,54]. This temperature tolerance becomes even more significant when talking about probiotics for veterinary use as feed supplements and, within this sector, aquaculture is the most challenging market, due to the extreme temperatures that are reached during the processing of fish feed. In fact, although a multitude of solutions and technologies have been developed to minimize the impact that temperatures can have, not only on the probiotic microorganisms, but also on the nutrients themselves, the possession of intrinsic mechanisms that allow them to face diverse challenges broadens the possibilities of use [55,56,57]. In this regard, the finding of several genes involved in increased resistance to suboptimal temperature conditions, both heat and cold (such as molecular chaperones GroES and GroEL, and several CSP), supports the probiotic potential of *L. cremoris* WA2-67 [58,59].

Furthermore, the replacement of fishmeal and fish oil by other more sustainable sources of protein and fat in feed production is one of the main challenges facing modern aquaculture. So far, vegetable sources have been the only real alternative at industrial level that has been able to reduce the inclusion of these ingredients [60]. However, a higher content of plant-based ingredients has meant an increase in the presence of undesirable non-starch polysaccharides (NSP) and phytate. Although xylanases are routinely added to feed, the presence of probiotic microorganisms that possess the genes for encoding xylanases may be of great interest in preventing the negative effects that these substances can have on fish health. [61,62]. Regarding the codification of amylase and lipases, in addition to its technological interest as a potential producer of these enzymes (cell factory), it is a probiotic characteristic occasionally present in LAB and, more specifically, in lactococci [63,64]. Furthermore, amylase, lipase and other digestive enzymes have been shown to contribute to improved nutrient assimilation in aquaculture, resulting in a lower feed conversion ratio (FCR) [65,66]. In this regard, α-amylase plays a crucial role in fish digestion of complex carbohydrates, such as glycogen and starch, which are common compounds in the feed of aquacultured species [67]. Therefore, the ability of fish to synthesize these enzymes, or their external supplementation through feed, will strongly determine their capacity to degrade and assimilate carbohydrates and proteins [68].

#### 3.2.3. Bacteriocin Production

**Figure 2:**
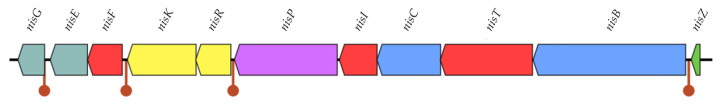
Prediction of the gene cluster encoding the production of NisZ in *L. cremoris* WA2-67 using the online webserver BAGEL v.4.0. (http://bagel4.molgenrug.nl/, accessed on 25 December 2021) [29]. Predicted terminators are shown as maroon line circle ends.

Bacteriocin mining, through the use of BAGEL v.4.0., identified three bacteriocinogenic genetic clusters in the genome of *L. cremoris* WA2-67. In node 5, the webserver predicted the presence of the genetic cluster encoding the production of the lantibiotic nisin Z (NisZ), which is the most widespread natural variant of Nisin A [16]. This cluster includes 11 genes, typically organized into four operons: *nisZ* (nisin structural gene), *nisBTCIP* (nisin maturation, immunity, and transport), *nisRK* (nisin regulation), and *nisFEG* (nisin immunity) (Figure 2) [69,70,71].

The *nisZ* gene encodes a NisZ biologically inactive precursor peptide of 57 amino acid residues, with a 23-residue N-terminal leader-sequence (pre-NisZ). The intracellular post-translational modifications of pre-NisZ require the existence of two membrane-associated enzymes, encoded by *nisB* and *nisC*, which catalyze the dehydration of serine and threonine residues and the formation of methyl-lanthionine or lanthionine rings, respectively [70,71,72]. Then, the fully modified but inactive NisZ is translocated through the cytoplasmatic membrane by an ATP-binding cassette (ABC) transporter, encoded by *nisT* [71,72], that forms a membrane associated complex with NisB and NisC. Finally, the leader peptide is cleaved off by a cell-membrane anchored subtilisin-like protease encoded by *nisP*, resulting in the mature and biologically active NisZ [71,73]. Furthermore, there are two mechanisms by which nisin-producing strains protect themselves against the toxicity of their own bacteriocin (self-immunity): (i) the lipoprotein encoded by *nisI*, and (ii) the ABC transporter and accessory proteins encoded by *nisFEG* [71,74]. Finally, NisZ biosynthesis relies on a two-component regulatory system, activated by NisZ, which consists of a sensor histidine kinase and a response regulator, encoded by *nisK* and *nisR*, respectively [71]. Additionally, the gene clusters of two previously described bacteriocins were identified: a bacteriocin of the garvicin Q family (node 10), first identified in *L. garvieae* BCC43578 [75], and a sactipeptide (node 13) related to the PqqA peptide cyclase of *Gluconobacter oxydans* [76]. In this regard, we have previously demonstrated that the knockout isogenic mutant strain *L. cremoris* WA2-67 Δ*nisZ*, lacking *nisZ*, does not exert antimicrobial activity against any of the tested indicator microorganisms [16]. Based on these observations it can be hypothesized that: (i) these bacteriocinogenic operons are not functional in *L. cremoris* WA2-67 (e.g., mutations or inactivation of the bacteriocin structural gene and/or genes involved in its regulation and/or transport) and/or (ii) that these bacteriocins are not biologically active under the experimental conditions and/or against the tested indicator microorganisms. Nevertheless, it is possible that the presence of these genetic clusters might be common in *Lactococcus lactis* subsp. *cremoris*. In this regard, the analysis of the reference strain, *L. cremoris* KW2 (RefSeq: NC_022369.1), also unveiled the presence of such genetic clusters. Therefore, further work would be necessary to proceed with: (i) functional analysis of the operon of these bacteriocins (e.g., identification of transcriptional promoters and terminators, identification of ribosome binding sequences and transcriptional regulators, transcriptomic analysis, etc.), (ii) evaluation of its antimicrobial activity against a larger number of indicator microorganisms, and (iii) in vitro synthesis and/or heterologous expression/secretion of these bacteriocins in various hosts, both prokaryotic and eukaryotic (e.g., *Escherichia coli* and *Pichia pastoris*).

#### 3.2.4. MGE (IS, Plasmids and Prophages)

The search through the ISfinder service revealed the presence of two true IS, that is, IS that obtain an e-value of 0.0 and in which each open reading frame (ORF) is recognized as a transposase [77] (Table 1).

After running an analysis using the PlasmidFinder web-tool, no plasmids were found in the *L. cremoris* WA2-67 draft genome (Table 1). This result is to some extent a positive probiotic characteristic, as plasmids can often carry antimicrobial resistance and virulence factor genes [31].

The Prophage Hunter web-service did not predict any prophage region in the WGS of *L. cremoris* WA2-67 (Table 1). Once again, this may reveal additional attractive traits, as some prophages can participate in cellular processes such as resistance to antibiotics, development of virulent characteristics, and new deleterious metabolic pathways [33,78].

#### 3.2.5. CRISPR/CRISPR-Cas

The presence of CRISPR-Cas systems, an adaptative immunity mechanism against the integration of exogenous DNA fragments, mainly MGE, was analyzed by the CRISPRCasFinder online program. In this regard, three sequences matched a positive prediction for the existence of CRISPR arrays. Nevertheless, when adjusting from default settings to hiding CRISPR arrays with an evidence level of 1 (1 being the lower evidence level out of 4) only one sequence remained, positioned in node 27, with an evidence level of 3 (Table 1). Furthermore, when the results were adjusted to hide sequences without the *cas* protein no matches were found. In this regard, when using the CRISPRCasFinder, program sequences with evidence level below 3 should be disregarded, as they indicate potentially invalid CRISPR arrays [34,79].

#### 3.2.6. Transferable Antibiotic Resistances

One of the most important characteristics for a bacterial strain to be proposed as a safe microorganism, and eventually as a probiotic, is the absence of transmissible antibiotic resistances, as they pose a threat to both animal and human health. Although LAB are generally classified with GRAS and/or QPS status, it is of the upmost importance to screen all potential LAB probiotic candidates for transferable antimicrobial resistances, as they can still act as reservoirs for antimicrobial resistance genes [8,80,81,82].

In this regard, the BLASTn search performed against the ResFinder tool v.4.1. database confirmed the absence of transferable and acquirable antibiotic resistances. Therefore, confirming the results previously observed that positively assessed the safety of *L. cremoris* WA2-67 [15].

#### 3.2.7. Virulence Factors

When assessing the potential use of LAB strains as probiotics, additional traits, such as virulence factors, should also be screened, both in vitro and in silico [83,84]. In this respect, the BLASTn search on the VirulenceFinder v.2.0.3 database found no matchings, confirming the lack of virulence factors. Once again, these results are in accordance with the in vitro safety assessment experiments previously performed for *L. cremoris* WA2-67 [15].

Moreover, the input of the PathogenFinder v.1.1. webserver predicted *L. cremoris* WA2-67 as a non-human pathogen microorganism. The prediction calculated the probability of being a human pathogen as 0.127, matching 0 and 220, pathogenic and non-pathogenic families, respectively.

## 4. Conclusions

The present manuscript highlights the importance of the in silico analysis of WGS of probiotic candidates, in particular *L. cremoris* WA2-67, which allows not only the detailed assessment of their safety and probiotic traits, but also the confirmation of previous in vitro and in vivo experimental results. Since WGS has become generally economically affordable, it is possible to assess multiple genetic machinery traits of a bacterial strain in a way that would not be sustainable and affordable using in vitro assays. In this regard, the WGS of *L. cremoris* WA2-67 allowed the analysis of the genetic cluster encoding production of NisZ, and the unexpected discovery of additional genetic clusters for the production of other two bacteriocins, namely, a member of the garvicin Q family and a sactipeptide. Moreover, the absence of both transferable antibiotic resistance determinants and genes encoding detrimental enzymatic activities and virulence factors demonstrates the safety of this strain. Furthermore, this work has shown several probiotic traits of *L. cremoris* WA2-67, such as the genetic enzymatic machinery for the production of tryptophan, other amino acids and vitamins, adhesion and aggregation factors, and stress adaptation mechanisms. Nonetheless, these probiotic characteristics, identified via in silico analysis, should be further assessed through experimental studies to confirm their expression and active production.

In summary, the data presented herein strongly support the future use of the bacteriocinogenic strain *L. cremoris* WA2-67 as a probiotic for aquaculture.

## Figures and Tables

**Table 1:** MGE (IS, prophages and plasmids) and CRISPR-cas systems identified in the genome of *L. cremoris* WA2-67, characterized in this work.

Analyzed Element	*L. cremoris* WA2-67
IS	IS similar/family/origin/length (bp)
	IS981/IS3/*Lactococcus lactis*/1224 IS-LL6/IS3/*Lactococcus lactis*/1254
Plasmids	ND ^a^
Active prophages	ND ^a^
CRISPR-cas systems ^b^	CRISPR spacers/cas genes/contig
	4/ND/27

^a^ ND: Not detected. ^b^ CRISPR-cas: clustered regularly interspaced short palindromic repeats—CRISPR associated protein.

## Data Availability

This Whole Genome Shotgun project has been deposited at DDBJ/ENA/GenBank under the accession number JAJONJ000000000. The version described in this paper is version JAJONJ010000000.

## Supplementary Materials

The following supporting information can be downloaded at: https://www.mdpi.com/article/10.3390/microorganisms10030521/s1, Table S1: Probiotic characteristics based on genome analysis.
