# Supplementary material for: Draft Genome Sequence of Lactococcus lactis Subsp. cremoris WA2-67: A Promising Nisin-Producing Probiotic Strain Isolated from the Rearing Environment of a Spanish Rainbow Trout (Oncorhynchus mykiss, Walbaum) Farm
Source: Microorganisms. 2022 Feb 28;10(3):521. doi: 10.3390/microorganisms10030521 (PMC8954438; doi:10.3390/microorganisms10030521)
Supplement: Supplementary file 1 [file microorganisms-10-00521-s001.zip › microorganisms-1552407-supplementary.pdf]

Table S1. Probiotic characteristics based on genome analysis.

Gene Function	Size (bp)	Contig
**Adhesion and aggregation**		
Enolase	1,257	1
Fibronectin-binding protein	1,623	12
Exopolysaccharides (EPS) biosynthesis		
EPS biosynthesis protein	909	2
Glycosyl transferase, group 1 family protein	-	-
Manganese-dependent protein-tyrosine phosphatase	-	-
Tyrosine-protein kinase EpsD	-	-
Tyrosine-protein kinase transmembrane modulator EpsC	-	-
Triosephosphate isomerase	759	2
AntiadhesinPls	-	-
Sortase A, LPXTG	753	2
ATP synthase ε chain	-	-
DNA polymerase III, ε related 3′-5′ exonuclease	-	-
Mucus-binding protein	-	-
**Vitamin biosynthesis**		
**Thiamine**		
Cytosine/purine/uracil/thiamine/allantoin permease family protein	1,293	10
HMP-PP hydrolase (pyridoxal phosphatase) Cof	-	-
Hydroxyethylthiazole kinase	753	7
Hydroxymethylpyrimidine ABC transporter: ATPase component	-	-
Hydroxymethylpyrimidine ABC transporter: substrate-binding component	-	-
Hydroxymethylpyrimidine ABC transporter: transmembrane component	-	-
Thiamin ECF transporter: substrate-specific component ThiT	549	1
Thiazole ECF transporter: substrate-specific component ThiW	-	-
Thiamin pyrophosphokinase	636	3
Thiaminase II involved in salvage of thiamin pyrimidine moiety		
Xanthine/uracil/thiamine/ascorbate permease family protein	1,422	3
**Riboflavin**		
3,4-dihydroxy-2-butanone 4-phosphate synthase	1,197	2
6,7-dimethyl-8-ribityllumazine synthase	465	2
ATP phosphoribosyltransferase	-	-
Diacylglycerol kinase	456	2
Diaminohydroxyphosphoribosylaminopyrimidine deaminase/5-amino-6-(5-phosphoribosylamino)uracil reductase	1,089	2
Hypothetical protein YebC	-	-
NADH dehydrogenase	624/1,260/2,004	6/8/8
N-terminal domain of CinA protein, C-terminal domain of CinA type S	-	-
Orotidine 5′-phosphate decarboxylase	714	12
Phosphoribosyl-AMP cyclohydrolase/Phosphoribosyl-ATP pyrophosphatase	639	7
Riboflavin kinase/FMN adenylyltransferase	924	2
Riboflavin synthase eubacterial/eukaryotic	648	2
Ribulose-phosphate 3-epimerase	651	4

Riboflavin ECF transporter: substrate-specific component RibU	621	7
Transcription termination protein NusB	978	5
tRNA pseudouridine synthase B	993	2
**Pyridoxin**		
D-3-phosphoglycerate dehydrogenase	1,197	5
Hypothetical NagD-like phosphatase	774	2
1-deoxy-D-xylulose 5-phosphate synthase	1,800/1,743	6/11
Pyridoxamine 5′-phosphate oxidase	378/507	4/4
Pyridoxine biosynthesis glutamine amidotransferase, glutaminase subunit	-	-
NAD-dependent glyceraldehyde-3-phosphate dehydrogenase	1,014/1,011	5/9
**Biotin**		
3-ketoacyl-CoA thiolase/Acetyl-CoA acetyltransferase	1,149/1,140	3/6
Acetoacetyl-CoA synthetase/Long-chain-fatty-acid–CoA ligase	-	-
Adenosylmethionine-8-amino-7-oxononanoate aminotransferase	-	-
Biotin ECF transporter: ATPase component BioM of energizing module	-	-
Biotin ECF transporter: substrate-specific component BioY	549/570	1/3
Biotin synthase	-	-
Biotin—protein ligase	753	3
Biotin operon repressor	972	3
Competence protein F homolog, phosphoribosyltransferase domain	651	2
Long-chain-fatty-acid–CoA ligase	1,245	3
Biotin carboxyl carrier protein of acetyl-CoA carboxylase	468	8
Biotin carboxyl carrier protein of methylcrotonyl-CoA carboxylase	-	-
Biotin carboxylase of acetyl-CoA carboxylase	1,368	8
Biotin carboxylase of methylcrotonyl-CoA carboxylase	-	-
**Folate**		
Dihydrofolate synthase/folylpolyglutamate synthase	1,284	2
GTP cyclohydrolase I type 1	1,050	2
Pantoate—β-alanine ligase	-	-
Aspartate 1-decarboxylase	-	-
2-amino-4-hydroxy-6-hydroxymethyldihydropteridine pyrophosphokinase	-	-
Dihydroneopterin aldolase	351	2
Dihydropteroate synthase	1,074	2
Cell division protein FtsH	2,088	1
Hypoxanthine-guanine phosphoribosyltransferase	552	1
tRNA (Ile)-lysidine synthetase	1,269	1
Para-aminobenzoate synthase, amidotransferase component	582	7
5-formyltetrahydrofolate cyclo-ligase	534	1
Dihydrofolate reductase	507	2
Thymidylate synthase	840	6
**Aminoacids metabolism**		
**Threonine**		
Biosynthetic aromatic amino acid aminotransferase α	-	-
Aspartokinase	1,353	8
Homoserine dehydrogenase	1,287	2
Aspartate aminotransferase	1,182	3

Aspartate-semialdehyde dehydrogenase	1,077	10
Homoserine kinase	891	2
Threonine synthase	1,491	4
**Tryptophan**		
Tryptophan synthase α chain	762	6
Tryptophan synthase β chain	1,206	6
Phosphoribosylformimino-5-aminoimidazole carboxamide ribotide isomerase	741	7
Para-aminobenzoate synthase, amidotransferase component	582	7
Isochorismatase	495	4
Indole-3-glycerol phosphate synthase	795	6
Anthranilate phosphoribosyltransferase	1,008	6
Anthranilate synthase, amidotransferase component	582	6
Anthranilate synthase, aminase component	1,371	6
**Methionine**		
Cystathionine γ-lyase	1,140	8
Cystathionine γ-synthase	1,089	4
S-adenosylmethionine synthase	1,200	3
Serine acetyltransferase	600	3
Homoserine O-succinyltransferase	960	4
Methionine ABC transporter ATP-binding protein	1,107	1
5-methyltetrahydrofolate—homocysteine methyltransferase	-	-
5,10-methylenetetrahydrofolate reductase	858	7
O-acetylhomoserine sulfhydrylase	1,281	1
SAM-dependent methlytransferase YrrT	621	2
**Leucine**		
3-isopropylmalate dehydrogenase	1,032	7
Isopropylmalate isomerase	-	-
2-isopropylmalate synthase	1,542	7
**Lysine**		
Aspartokinase	1,353	8
Lysine ε-oxidase	-	-
4-hydroxy-tetrahydrodipicolinate synthase	894	10
4-hydroxy-tetrahydrodipicolinate reductase	777	6
Aspartate-semialdehyde dehydrogenase	1,077	10
Diaminopimelate decarboxylase	1,260	7
N-acetyldiaminopimelate deacetylase	1,131	1
2,3,4,5-tetrahydropyridine-2,6-dicarboxylate N-acetyltransferase	771	1
N-acetyl-L,L-diaminopimelate aminotransferase	1,176	1
**Cysteine**		
Cysteine synthase	933/921	1/8
Phosphoadenylyl-sulfate reductase (thioredoxin)	315	11
Cysteinyl-tRNA synthetase	1,347	3
Sulfite reductase [NADPH] hemoprotein β-component	-	-
Sulfite reductase [NADPH] flavoprotein α-component	-	-
**Histidine**		
Phosphoribosyl-AMP cyclohydrolase	639	7
Imidazole glycerol phosphate synthase, regulatory subunit	780	7
Imidazole-glycerol-phosphate dehydratase	600	7
Histidinol dehydrogenase	1,308	7
ATP phosphoribosyltransferase	957	7

Histidinol-phosphate aminotransferase	1,071	7
Histidinol phosphatase	786	7
**Arginine**		
N-acetyl-γ-glutamyl-phosphate reductase	1,023	8
N-acetylglutamate synthase	1,188	8
N-acetylglutamate kinase	852	8
N-acetylornithine aminotransferase	1,134	8
Ornithine carbamoyltransferase	1,065/1,002	4/8
Argininosuccinate synthase	1,197	1
Argininosuccinate lyase	1,380	1
Arginine pathway regulatory protein ArgR	459	4
**Production of lactic acid**		
D-lactate dehydrogenase	-	-
L-lactate dehydrogenase	897/945/972/978	1/1/2/12
**Active metabolism**		
Poly (glycerol-phosphate) α-glucosyltransferase	1,512	8
β-1,3-glucosyltransferase	933/960/981/1,014	2/4/4/4
Xylose isomerase domain protein TIM barrel	-	-
**Enzyme production for food digestion**		
α-amylase	1,575	11
Lipases	840/564/846/774	1/3/6/7
Serine protease extracellular	-	-
Phytase	-	-
Cellulase	-	-
Xylanase	1,134	7
**Stress adaptation / host gastrointestinal tract adaptation**		
**Temperature tolerance**		
Cold shock protein (CSP) family	198/201/201/201/201/201	1/2/5/5/5/5
Heat shock protein DnaJ	-	-
Heat shock protein DnaK	-	-
Heat shock protein HtpX	-	-
Heat shock protein Hsp33	-	-
Heat shock protein GrpE	540	2
Heat shock protein Hsp70	-	-
Small heat shock protein	-	-
Ribosome-associated heat shock protein	270	1
Co-chaperonin GroES (heat shock protein)	285	1
Molecular chaperone GroEL (heat shock protein)	1,629	1
**Acid tolerance**		
ATP synthase subunit a	714	3
ATP synthase subunit b	507	3
ATP synthase subunit c	216	3
ATP synthase α chain	1,503	3
ATP synthase β chain	1,410	3
ATP synthase γ chain	870	3
ATP synthase δ chain	528	3
PTS system, cellobiose-specific IIC component	1,338/1,485/1,308/1,356/1,419	1/1/2/6/8
ATP-dependent Clp protease ATP-binding subunit ClpB	2,604	6
ATP-dependent Clp protease ATP-binding subunit ClpC	2,451	5
ATP-dependent Clp protease ATP-binding subunit ClpE	2,247	5
ATP-dependent Clp protease ATP-binding subunit ClpP	600	5

ATP-dependent Clp protease ATP-binding subunit ClpX	1,236	2
Glucose-6-phosphate isomerase	1,347	13
GTP pyrophosphokinase	681/675	1/2
Pyruvate kinase	1,509	12
**pH tolerance**		
Sodium-proton antiporter	2,043/1,140/1,950	1/4/7
Alkaline shock proteins	402	5
F0F1-ATPase	-	-
**Bile salts tolerance**		
Choloylglycine hydrolase	-	-
Bile salt hydrolase	-	-
Glucosamine-6-phosphate deaminase	714/708	3/6
CTP synthase	1,608	1
DamX	-	-
**Osmotic stress tolerance**		
Glycine betaine		
ABC transporter, ATP-binding protein OpuAA	918	2
ABC transporter, ATP-binding protein BusAA	1,224	6
ABC transporter, substrate binding protein and permease (BusAB.1/BusAB.2)	1,722	6
ABC transporter, binding protein and permease (OpuAB/OpuAC)	1,503	2
Manganese-dependent inorganic pyrophosphatase	945	3
**Oxidative stress tolerance**		
Catalase	-	-
Thiol peroxidase	483	1
Glutathione reductase	429/825	2/2
Glutathione peroxidase	474	12
NADH peroxidase	-	-
NADH oxidase	1,341/1,332	1/4
NADH flavin oxidoreductase	-	-
Haloperoxidase	-	-
Iron dependent peroxidase	-	-
Thioredoxin reductase (NADPH)	927/966	2/11
Pyruvate oxidase	1,725	4
Dihydroorotate oxidase	-	-
Cadmium-/manganese-transporting P-type ATPase	-	-
Manganese ABC transporter	714/840/987	12/12/12
